# Aspects that facilitate access to care for viral hepatitis: An evaluative research

**DOI:** 10.1590/1516-3180.2023.0078.R1.29112023

**Published:** 2024-03-11

**Authors:** Josué Souza Gleriano, Carlise Krein, Lucieli Dias Pedreschi Chaves

**Affiliations:** I PhD. Nurse, Adjunct Professor, Department of Nursing, Faculty of Agricultural, Biological, Engineering and Health Sciences, Universidade do Estado de Mato Grosso (UNEMAT), Tangará da Serra (MT), Brazil.; II Msc. Nurse, Department of General and Specialized Nursing, Ribeirão Preto School of Nursing, Universidade de São Paulo (USP), Ribeirão Preto (SP), Brazil.; III PhD. Nurse, Associate Professor, Department of General and Specialized Nursing, Ribeirão Preto School of Nursing, Universidade de São Paulo (USP), Ribeirão Preto (SP), Brazil.

**Keywords:** Comprehensive health care, Hepatitis, viral, human, Health services, Health services accessibility, Patient acceptance of health care, Governance, Regionalization, Evaluative research in health, Management of health systems and services, Health management, Unified health system

## Abstract

**BACKGROUND:**

Viral hepatitis is a major public health concern worldwide.

**OBJECTIVES:**

This study aimed to analyze the factors that facilitate access to care for viral hepatitis.

**DESIGN AND SETTING:**

Using a sequential mixed method, this evaluation research was conducted in the state of Mato Grosso, Brazil.

**METHODS:**

Mapping of references and selection of regions were made based on the quantity and heterogeneity of services. The stakeholders, including the managers of the State Department of Health and professionals from reference services, were identified. Nine semi-structured interviews were conducted using content analysis and discussions guided by the dimensions of the analysis model of universal access to health services.

**RESULTS:**

In the political dimension, decentralizing services and adhering to the Intermunicipal Health Consortium are highly encouraged. In the economic-social dimension, a commitment exists to allocate public funds for the expansion of referral services and subsidies to support users in their travel for appointments, medications, and examinations. In the organizational dimension, the availability of inputs for testing, definition of user flow, ease of scheduling appointments, coordination by primary care in testing, collaboration following the guidelines and protocols, and engagement in extramural activities are guaranteed. In the technical dimension, professionals actively commit to the service and offer different opening hours, guarantee the presence of an infectious physician, expand training opportunities, and establish intersectoral partnerships. In the symbolic dimension, professionals actively listen to the experiences of users throughout their care trajectory and demonstrate empathy.

**CONCLUSIONS:**

The results are crucial for improving comprehensiveness, but necessitate managerial efforts to enhance regional governance.

## INTRODUCTION

Viral hepatitis is characterized by liver inflammation caused by an infection with five types of viruses.^1^ It is considered a significant global public health problem, necessitating the implementation of health management strategies.

Worldwide, approximately 257 million people living with chronic hepatitis B virus infection and 71 million people with hepatitis C virus infection are unaware of their condition. Approximately 57% of liver cirrhosis cases and 78% of primary liver cancer cases are attributed to hepatitis B and C virus infections.^2^ In Brazil, from 2000 to 2021, 718,651 patients were diagnosed with human viral hepatitis. Of them, 168,175 (23.4%) had hepatitis A, 264,640 (36.8%) had hepatitis B, 279,872 (38.9%) had hepatitis C, and 4,259 (0.6%) had hepatitis D. From 2000 to 2020, 82,169 deaths due to fundamental causes and linked to hepatitis types A, B, C, and D were reported. The distribution of these deaths was as follows: 1.6% for viral hepatitis A, 21.3% for hepatitis B, 76.2% for hepatitis C, and 0.9% for hepatitis D.^3^

Following the World Health Assembly’s recommendation, a global initiative was launched to advance the elimination of hepatitis. This movement aims to achieve a 90% reduction in the new infection rate and a 65% reduction in the mortality rate by strengthening access to diagnosis and treatment to stop virus transmission.^4^ In the Americas and Caribbean region, the Pan American Health Organization (PAHO) has advocated for equitable access to preventive and diagnostic care, emphasizing an integrated response from the Health System (HS) to strengthen case surveillance.^5^ In recent decades, notable progress has been observed in the adoption of preventive measures through immunization. However, the primary emphasis has been on the treatment aspect.^6^ Strengthening measures to eliminate viral hepatitis is part of the 2030 Agenda.^7^

In Brazil, following the establishment of the National Program for the Prevention and Control of Viral Hepatitis (NPVH), in the Unified Health System (SUS), various coping strategies that permeate disease prevention and diagnosis, and guidelines for the organization of care networks have been implemented.

Considering the extensive scientific research on the clinical aspects and treatment of hepatitis, along with the guidelines provided by the 2030 Agenda for Sustainable Development^7^; the need to enhance access to the diagnosis and treatment of viral hepatitis; and the implications for the organization of healthcare, this study aimed to determine the aspects that facilitate access to care for viral hepatitis. The results are anticipated to make valuable contributions to understanding access to care, influencing health management demands for the organization of care networks and assistance across various points of care from the perspective of prevention, diagnosis, and treatment.

## OBJECTIVE

This study aimed to analyze the factors that facilitate access to care for viral hepatitis.

## METHOD

This evaluative research^8^ utilized the mixed sequential method.^9,10^ This study^11^ was conducted in the state of Mato Grosso, which comprised 6 macro-regions and 16 Health Regions (HRs). This location was selected owing to its adherence to decentralized management and a regionalization process.^12^

In the quantitative stage, data from the National Register of Health Establishments (NRHE) in the second half of 2020 were reviewed to identify reference services for the treatment of viral hepatitis. The HR demonstrating a higher quantity and heterogeneity of referral services were selected for in-depth analysis, signifying its capacity to provide a comprehensive range of health actions and services. The South Mato Grosso HR not only fulfilled the criteria of having a high population density but also boasted seven dedicated services for the treatment of viral hepatitis, with six actively participating in the study. The service allocated to the penitentiary was an exception, as its organizational conditions for attention require a specific approach.^13^

In line with the criteria employed for HR selection, stakeholders were identified based on the following inclusion criteria: individuals responsible for overseeing health management in the viral hepatitis sector of the State Health Secretariat of Mato Grosso (SHS-MT) and professionals responsible for technical services, specifically the Testing and Counseling Center (TCC) and/or Specialized Assistance Service (SAS), with a minimum of 6 months of active service. Professionals who were absent from their duties for any reason were excluded.

Individual semi-structured interviews were conducted to collect data. The interview script was submitted for face validation and pre-test application. Participants were initially contacted via email and phone. Upon formal acceptance by email along with the submission of the signed informed consent form, the interview was scheduled on a digital platform (WhatsApp, Google Meet, or Zoom) at a mutually agreed upon time.

In addition to the script, a vignette was used to facilitate participant engagement^14^. The interviews lasted for an average of 50 min and were conducted by the lead researcher between August 2020 and January 2021. The material was transcribed, and participants were identified by the letter P, followed by an Arabic numeral. Thematic analysis and data systematization were performed,^15^ with the content grouped into cores of meaning. Analysis categories were defined, with a focus on aspects that facilitate attention to viral hepatitis.

Subsequently, to highlight these aspects, the category was compared within the dimensions of the Analysis Model: universal access to health services.^16^ To validate the accuracy of the data and ensure the appropriateness of the analyses in capturing the intended messages from the key informants, they were re-invited to provide feedback using Google Forms. An agreement rate of >70% on the dimensions was set as the acceptance criterion.

The Analysis Model Universal access to health services^16^ was adopted to structure the presentation of results and foster discussion at the interface. This study was approved by the Research Ethics Committee (CAAE: 01481918.0.0000.5393) and the co-participating institution (CAAE: 01481918.0.3001.5164).

## RESULTS

Twelve key informants were considered eligible; nine participated in the study, specifically technical professionals from the state management of the SHS involved in coordinating the NPHV and the reference services of the HR. The participants predominantly consisted of individuals with permanent employment status (77,8%), individuals with a service tenure exceeding 3 years (88,8%), women (77,7%), individuals of white ethnicity (66,6%), individuals aged >50 years (44,4%), individuals with a nursing background (44,4%), and individuals who received postgraduate training (88,8%).

Table 1 presents the main findings of the analysis of access dimensions. The level of agreement between the participants and the analyses was higher than 90%.

**Table 1:** Aspects that facilitate access to care for viral hepatitis, according to the dimensions of analysis, Mato Grosso, 2021

ANALYSIS DIMENSION: POLICY		
Aspects analyzed	Key results	Key informant speech strata
Process monitoring (NPHV) Agreement between the instances (state and municipal)	**- Federal scope:** - Brazilian Ministry of Health acted as a policy inducer that favored the decentralization and implementation of services and increased testing offer.	*“The Ministry encouraged the implementation of TCC. It was important to have this service here”.* P4 *“It seems that the Ministry has been providing opportunities for the decentralization of services”.* P8 *“The Kits are just ask and they come from the Ministry of Health, this has no cost to the municipality”.* P3
	**- Inter-municipal agreement:** - The health consortium has been the management instrument for agreement favoring access.	*“The consortium was the right strategy. In logistics, the physician attends here at the SAE twice a month for the references of the municipalities. They don’t have a hard time buying the consultation; it’s not an expensive consultation.”* P6 *“With the consortium we were able to hire the specialist.”* P5
**ANALYSIS DIMENSION**: **ECONOMIC-SOCIAL**		
Investments in the public network by sphere of power and level of complexity. Social, economic, cultural, and physical barriers	**Investment in the health sector has aligned improvements in infrastructure and resources to reduce barriers:** - Recognition of the epidemiological scenario by management, given the increase in reported cases of human immunodeficiency virus infection and sexually transmitted infections, motivated the creation of reference services such as TCC and SAS. - The infrastructure and human resources framework in the SAE that is used as a reference for attention to hepatitis in the health region was considered satisfactory in relation to the other services in the state.	*“The number of people being treated for HIV has increased. So it was proposed to create the TCC to advance testing”.* P4 *“Last year (2020) we started a conversation to implement an SAE in the municipality. With the project we moved to a better physical space ”.* P7 *“Look, if you are going to make a comparison with other municipalities, we are fine. For a while I got a room for myself”.* P3 *“The SAE infrastructure, the multi-professional team is a differential in care. The ease of collecting the material is what gives us strength, we have the laboratory and we collect almost weekly viral load. We reduce the waiting time of the patients, at the latest it is ten days. We also collect for genotyping. The result today is a quick result, before it was 30 or more today from ten to fifteen days”.* P6
	**Ensuring attention from the perspective of user needs:** - The attitude of the municipal manager and the local economy contributes to the displacement of the users to the consultation, covering medications outside the REMUNE (Municipal List of Essential Medicines) and examinations that are not included in the agreement list.	*“Even if the patient has to move from the municipality, transportation is provided and in some cases OHT (Out-of-Home Treatment) has already been offered. The patient has the facility of the examination, of the medication, including those that are out of REMUNE, because sometimes the infectious disease physician passes another medication and the secretary provides it. We had a hepatitis C patient who was given a OHT for her to have tests, which were not in the agreed list in the consortium, she did it in a private service. ”* P8
**ANALYSIS DIMENSION**: **ORGANIZATIONAL**		
Entry Door Attendance flow Geographical barriers, Regulation Reference Counter-reference Evaluation	**User flow in the regulation system:** - There are no limitations to the offer of testing in TCC and SAE services. - There is a defined flow to the users as soon as they receive the diagnosis. - The expansion of the offer of testing through Primary Health Care and the organization of flows in the municipality is a favorable point. - It is easy to schedule appointments through the consortium. - Female population deprived of liberty has partnerships for actions with the SAE of Rondonópolis. - The care protocol for pregnant women with a flowchart that guides professionals favors the organization of care and access to prevention of vertical transmission.	*“If the user arrives here, there is a test, if he goes to a basic unit and there is no test there, just forward him here and I will attend him.”* P4 *“Once this patient is diagnosed, he has a right way.”* P5 *“Quick test that gave reagent already has a right way”.* P8 *“We have five basic health units here, the rapid test is offered in the units and in the municipal hospital”.* P9 *“All FHS (Family Health Strategy) can perform the test, the nurse is trained. This goes a long way in increasing access to testing”.* P*4* *“The consortium calls and says that there is a patient who is sick; we tell the patient that we can manage to put him in the consultation here. Sometimes it puts this user on the agenda of Rondonópolis, but our concern is that this patient suffers as little as possible and that he has access to medication as soon as possible”.* P6 *“For pregnant women, the flow is easier because there is a protocol and all professionals already follow. The quick test is done on the unit, this makes it easier to guide the attention network, it doesn’t get so loose”.* P5 *“In the SAE we have a partnership with the female penitentiary, with actions there for screening and diagnosis and treatment. Already the male penitentiary they have a team of physicians and nurses there, they seek the SAE more to seek the test and also the medications”.* P5
**ANALYSIS DIMENSION**: **TECHNICAL**		
Welcoming Bond Competence Ability Autonomy Commitment Shared therapeutic project Team and user Quality of care	**Strategies to promote welcoming in the reference service:** - Scheduling various time slots at the CTA/SAE is a strategic approach. - Expanding extramural actions enhances testing. - Having an infectious disease physician in the network was recognized as favorable for facilitating diagnosis and treatment. - Professional training within the care network was considered an opportunity to advance in providing testing.	*“I give priority to attending in the morning, but if the patient comes in the afternoon, I attend. You have to consider who works, the sex workers who sleep in the morning”.* P3 *“We do a lot of lectures, this region is a farm, so in SIPAT (Internal Week for the Prevention of Accidents at Work) weeks one of the themes is HIV and viral hepatitis, and in addition, lectures go to schools. The lecture is the key car in the municipality, because when we go it is clear that people do not know about hepatitis”.* P4 *“We have an infectious disease physician in the unit, this is a gain; many SAE do not have. In the past he attended once a week, today he attends twice. He attended 15 patients; today he attends 20 each day, so there are 40 vacancies weekly. His schedule for other municipalities is 30 vacancies. The chance of treatment being faster and also right is higher with a specialist”.* P6 “There are few capacities, but they help to stay on top of the new protocols”. P5
	**Cross-sector alliances:** - - Partnerships between health services and social media, communication channels, and third-sector institutions have helped promote the prevention of hepatitis in society.	*“We have partners like the Catholic Church, AIDS pastoral, partnerships with higher education institutions, with television and radio media. We do interviews talking about epidemiological situation, talking about the importance of prevention and testing. Partnership with Non-governmental organizations that represent LGBT (Lesbian, Gay, Bisexual and Transgender groups and sex workers are intermediaries to get us there”.* P7 *“We partner with Rotary for campaigns, especially for hepatitis C. When you do actions with partnerships you can even reach a target audience”.* P8
	**Professional commitment:** - The proactive attitude of the professionals and their commitment to work result in greater benefit to access. - Efforts by professionals to assist the users in their demands increases adherence to treatment.	*“A couple of years ago I started talking to managers about the importance of better structuring the operation and creating a SAE, equipping and putting more professionals”.* P7 *“Our social worker does a very detailed job. It is a facilitator for hepatitis patients, especially in scheduling appointments and exams, in medication processes, with documentation and checking so that it works there in the removal of high-cost medications of the pharmacy”.* P5 *“The physician gives us a lot of access, we talk to him to solve a problem of a patient that appears, he opens a schedule and we manage to fit in”.* P6 *“Sometimes we have leftover HIV medication, because the patient dies and the medication is returned here, so if you have Tenofovir it stays as leftover in the pharmacy and we deliver it. So we stood between the cross and the sword, between doing right or doing wrong, but at that moment if we have leftovers here we will offer, the physician prescribes and we release, it is not the ideal way, but it is also not ideal to leave him in this situation if I have medication here”.* P5
**ANALYSIS DIMENSION**: **SYMBOLIC**		
Culture Beliefs Values Subjectivity	**The voice of the user after accessing the service mobilizes to qualify the assistance:** - The users’ experience in the utilization of the health service and the manner in which they communicate their management and professional interactions have contributed to the expansion of specialized reference services.	*“The patient has a voice, he talks about his difficulties. Although we are close to Rondonópolis, moving there and here is not easy. So he comes and says, when he has an opportunity, he takes it to the board or to the secretary; it helps us to show the manager the need to qualify the service”.* P7 *“The idea of bringing an SAE was due to complaints from patients about the times they had to go beyond the conditions of our roads and the wear and tear with the trip”.* P9
	- **Profile of the professional to favor access**: - The professional’s ability to empathize in serving the vulnerable population is an essential factor in understanding the approach to hepatitis in different territories.	*“For the service to walk, you need to identify yourself a lot with work, you have to like it, you have to break down prejudices, taboos. Example: we have a very large flow of homosexuals, sex workers and if suddenly you are a very closed professional, you may not be able to do the actions that are viable”. P4*

In the political dimension, the implementation of guidelines and financial support from the Ministry of Health (MH) favored the decentralization of services and increased testing (P3, P4, and P8). Simultaneously, the regional organization process, facilitated through an agreement with the Intermunicipal Health Consortium (IHC), ensured access (P5 and P6).

In the economic-social dimension, the summary of results includes the contributions of public sector investment toward reducing geographical barriers and expanding services in HRs (P3 and P4). This includes ensuring the user’s transfer to the reference service through an OHT instrument (P8) and investing in the human resources framework within the health sector of the reference service (P6 and P7).

The findings suggest that in the organizational dimension, providing testing in reference services is not challenging, especially with the expansion of testing offered by PHC (P4). There is a defined flow and rapid forwarding response to users with a positive diagnosis (P5 and P8). The use of IHC ensures appointment scheduling (P6), taking into account the initiatives of the reference service involving the prison sector (P5). Lastly, the organization of the care network flow is facilitated through the implementation of care protocols (P5).

In the technical dimension, the flexibility of TCC/SAS in offering different service hours was recognized as strategic (P3). Extramural actions were deemed essential for enhancing testing (P4). Partnerships with social media, communication channels, and third-sector institutions were identified as crucial for amplifying extramural actions (P7 and P8). The presence of an infectious disease physician in the referral service was considered a differentiating factor in case management, even facilitating access to treatment (P6). Training and updating for teams were emphasized (P5). Lastly, the proactive posture of referral professionals in organizing the service and providing maximum efficiency to treatment and monitoring was highlighted (P5, P6, and P7).

In the symbolic dimension, the results converge on the significance of municipal health management listening to the user regarding their therapeutic itinerary during (P7 and P9) and demonstrating empathy in serving the users within the service (P4).

## DISCUSSION

In the political dimension, the inclusion of hepatitis in the Sustainable Development Goals (SDG) has played a crucial role in emphasizing the significance of this disease within health systems. The challenge lies in mitigating inequalities in access to hepatitis care and ensuring treatment, considering the diverse social disparities present in health systems.^17^ To formulate public health policies tailored to the reality of HS, incorporating care models that streamline service provision and enhance access, it is necessary to establish indicators that support decision-making.^18^

The collective management guided by the coping model adopted in Europe serves as an exemplary experience for achieving micro-elimination by 2030.^19^ Low- and middle-income countries encounter challenges related to unequal access. However, Egypt, Georgia, Rwanda, and Mongolia have made notable progress by implementing targeted strategies aimed at serving priority groups.^20^ These successful experiences are linked to the expansion of infrastructure, the implementation of comprehensive public policies for testing and treatment, the systemic integration of various services, and the application of concerted efforts to reduce the cost of medicines in these countries.^20^

In Brazil, the MH initiatives are outlined through the NPVH. Since its inception, the program has established guidelines that emphasize prevention, surveillance, and assistance across various health services, including the organization, regulation, monitoring, and evaluation of the program’s actions. Since 2008, in technical collaboration with the World Health Organization (WHO), goals have been set to address this disease in Brazil.^21^ In terms of prevention, vaccination has been incorporated into the SUS immunization schedule, indicated for different population groups and age categories. This culminated in 2016, with expansion to the entire population, regardless of age.^22^

In the SUS, from 2005 to 2010, there was a noticeable expansion of the care network for testing and counseling of hepatitis B and C. This expansion was supported by Clinical Protocol of Therapeutic Guidelines publications and involved the incorporation of direct antiviral agents (DAAs), recognized by PAHO as a significant advancement in the care of patients with hepatitis C.^4,23^ Since 2020, to facilitate the expansion of access and adherence to treatment, the MH has enabled the migration of hepatitis drugs from the Specialized Component to the Strategic Component of Pharmaceutical Assistance.^24^

To enhance access, the Brazilian MH published Ordinance 1.537/2020 MH/GM,^24^ which underscores the necessity for greater coordination between the NPVH and the Health Surveillance Secretariat. This collaboration involves shared actions with the Secretariat of Primary Health Care, Secretariat of Specialized Health Care, National Health Foundation, National Health Surveillance Agency, and Secretariat for Science, Technology, and Strategic Inputs. Technical note number 369/2020 CGAHV/DCCI/SVS/MS was published in order to support the decentralization of testing and treatment through the involvement of nurses.^25^ Municipal health management has used the IHC to ensure access to specialty centers for confirmatory tests and the initiation of treatment. The IHC operates based on the logic of political-economic cooperation with territorial agreements, aiming to minimize bureaucratic processes and interfederal barriers.^11,26^

In the economic-social dimension of access, there is consensus that advancing policies to combat hepatitis can generate an economic benefit.^27^ The results demonstrate a strong emphasis on epidemiological aspects to expand services, primarily propelled by the robustness of the HIV program. Despite progress in decentralizing SAS-type services, HR care remains centralized in the regional reference service. Participants rarely explored this dimension, possibly due to the centrality of the treatment and the distant involvement of the state program management in coordinating the economic and social aspects of HR.

To advance regionalization, it is necessary to consider the criteria that can organize attention through regional planning. The European Union has prioritized organizing attention based on epidemiological data that subsidize referencing and creating services that offer focal actions to specific populations and risk groups to achieve microelimination.^28^

One aspect impacting any HS is the provision of treatment, with economic considerations arising from the high cost of medications. To expand guidelines that strengthen access, it is necessary to analyze costs. Alongside this, an investment strategy can be implemented to estimate treatment expenditures, recommending centralized purchasing supported by clinical practice guidelines.^29^ This approach, implemented in Colombia, resulted in a reduction of more than 90% in prices.^30^ In Brazil, a country with a universal public system playing a significant role in the distribution of hepatitis treatment, this practice has been adopted since 2006.^31^ It has facilitated the technological incorporation of treatment, particularly for hepatitis C treatment.^32^ Since 2014, access to treatment with DAA has been guaranteed, despite the high cost.^31^

In low- and middle-income countries, the number of studies addressing the costs and continuous follow-up of users from hepatitis diagnosis to cure is low.^33^ This limitation poses a challenge for hepatitis control policies in countries confronting significant social inequalities and dealing with fiscal austerity on the political agenda. The Brazilian MH has developed an economic model aligned with strategies considered potential to achieve the goals estimated by the WHO. This initiative aims to support an analysis of the costs associated with eliminating hepatitis C in the country by 2030.^34^

In addition to the infrastructure of the referral service, the participants identified professionals in the services and training for actions as crucial factors that enhance the possibility of advancing access. Allowing users to access reference services with OHT support ensures access and contributes to the realization of the principle of universality, promoting equity.^35^ However, the economic and logistical aspects of care networks have weakened, in many cases, the guarantee of OHT.^36^

In the organizational dimension, it is perceived that in HRs, a flow based on positive testing is defined, despite being centralized and requiring intersectoral negotiations. In light of this finding, health services should analyze the geographical barriers that may hinder users’ access.^37^

In regional organizations, analyzing strategic points for the dissemination of actions and services, with the support of specialist physicians in decentralized teams, increases the likelihood of improving the hepatitis program^38^. For this improvement to occur, effective professional communication is essential, which involves a multi-professional team to facilitate access in the territory, especially for priority groups.^39^

The regulation of care and care management are important in the work process. It is essential to have PHC in place to identify people individuals susceptible to diagnosis, particularly in the context of decentralizing hepatitis treatment^24^ and establishing matrix support for PHC teams. In SUS, the importance of PHC and the matrix support for its teams has been discussed since the creation of the NPHV^40^. Since 2021, new investments have been allocated to materialize this proposal, with initiatives aimed at enhancing the competence of clinical and pharmaceutical care professionals and fortifying the care pathway by formulating guidelines for the diagnosis, treatment, and referral of patients.^11^

The TCC in municipalities can strengthen the adoption of testing practices in PHC, support professional training, monitor referred cases, and promote a more organized user flow.^12^ In this context, it is essential to minimize the barriers to accessing treatment, especially access to medications that affect adherence. Implementing flexible documentation, monitoring support, and incorporating strategies for screening side effects and drug interactions are potential approaches for collaborative teams.^41^

In the technical dimension, positive aspects of access include organizing the work process to enhance reception, implementing measures to expand testing, and utilizing strategies to disseminate information about the disease. To overcome social, economic, cultural, and physical barriers, it is necessary to coordinate actions that integrate health services with rapid response flows in priority locations that serve socially vulnerable populations, especially those marginalized by HS.^24^ The support of specialists extends the connection with users in the territory and allows the dissemination of educational resources through multipliers.^44^ Personalization of attention to the users’ problem strengthens monitoring and increases the acceptability of treatment.^45^

To align with the WHO Hepatitis Testing Guidelines, various strategies are recommended, such as integrated HIV testing, use of social media to promote acceptance of tests, workplace testing, utilizing emergency departments for testing, directing professionals with notifications in electronic medical records of high-risk patients for testing, and expanding specific services in different populations, such as injecting drug users, prisoners, other high-risk groups, migrants, and relatives of people living with hepatitis B or C.^46^ For users born between 1945 and 1965,^47^ the study used personalized invitations through letters , which successfully increased the test response rate.

Involving the community is an essential strategy in planning actions that aim to advance the elimination of hepatitis.^27^ To achieve this, establishing partnerships with non-governmental organizations (NGOs) and providing training for a multidisciplinary approach in collaboration with civil society enhance the effectiveness of actions.^48,49^ Investing in a multidisciplinary approach to hepatitis care is a recent and rapidly evolving field. Care management that considers dynamic and collaborative practice approaches is likely to strengthen access to testing, diagnosis, and treatment adherence.^50^

In addition to performing testing, ensuring the accuracy of the diagnosis, and training professionals to make appropriate requests and interpret tests correctly for treatment, focusing on hepatitis necessitates a multimodal approach. This involves the use of technology resources and coordination of care.^41^ For this, managers can use electronic registration banks of health services to subsidize analyses that guide the selection of strategies.^51^

Nurses are highly effective professionals trained to assume the role of Hepatitis Clinical Nursing Consultants. HepCare Europe considers the role of this professional as crucial, serving as a mediator of communication between levels of care, from prevention to treatment adherence.^49^ In Australia,^52^ New Zealand,^53^ Baltimore,^54^ and the United States, the nurses-led strategy has demonstrated success, suggesting the optimization of health resources.

In the symbolic approach, prioritizing listening and providing opportunities for users to share their care experiences in the therapeutic itinerary justifies the increased decentralization of services in HR. Thus, understanding cultural aspects helps health services design actions based on proposals that can be more welcoming in different territories.^55^ The communication process that takes place in the professional-user relationship plays a key role in adherence to the recommendations and treatment of hepatitis.^56^

## CONCLUSIONS

In terms of the factors promoting access, the political dimension highlights the incentive of decentralizing services and the use of IHC in the agreements. In the economic-social dimension, the expansion of reference services, supported by public resources, involves the transportation of users to consultation and treatment centers, incorporating medications beyond the REMUNE, and covering examinations that are not included in the agreement list. In the organizational dimension, ensuring professionals’ confidence in the availability of testing supplies, establishing a clear flow for diagnosed users, and facilitating appointment scheduling are key considerations. Although the organization of care by PHC is beneficial, it necessitates the formulation of guidelines and protocols in collaboration with extramural activities through partnerships with the TCC/SAS, as exemplified in the prison context. In the technical dimension, the commitment of professionals to the service, offering diverse time slots at the TCC/SAS to meet the user’s demand, having an infectious disease physician in the network, and providing professional training, in addition to inter-sectoral partnerships, play crucial roles. In the symbolic dimension, listening to users’ experiences during the care trajectory and demonstrating professional empathy are emphasized.

Analysis of the participants’ discourse did not reveal solid strategies from state coordination guidelines that focused on testing indigenous populations, quilombolas, rubber tappers, artisanal fishermen, riverside dwellers, gays, men who have sex with men, transvestites and transsexuals, people who use drugs, the homeless, and those deprived of freedom. In HR, only two services cited actions with intersectoral partnerships to expand testing to sex workers. This situation highlights the lack of criteria aligned with political guidelines, in addition to the low coordination of actions in health services.

On the contrary, if a discourse is based on the manager’s concern with the goals established for the elimination of hepatitis C, the intensity of the provision of actions and services in the SUS care network can be reevaluated from the health promotion to risk and damage control perspectives.
